# Misdiagnosis of Rheumatoid Arthritis in a Long-Term Cohort of Early Arthritis Based on the ACR-1987 Classification Criteria

**DOI:** 10.2147/OARRR.S372724

**Published:** 2022-09-22

**Authors:** Monica Leu Agelii, Ingiäld Hafström, Björn Svensson, Sofia Ajeganova, Kristina Forslind, Maria Andersson, Inger Gjertsson

**Affiliations:** 1 Department of Rheumatology and Inflammation Research, Institute of Medicine, Sahlgrenska Academy, Gothenburg University, Gothenburg, Sweden; 2 Department of Medicine Huddinge, Division of Gastroenterology and Rheumatology, Karolinska Institutet, Karolinska University Hospital, Stockholm, Sweden; 3 Department of Clinical Sciences Lund, Section of Rheumatology, Lund University, Faculty of Medicine, Lund, Sweden; 4 Department of Clinical Sciences, Rheumatology Division, Universitair Ziekenhuis Brussel, Vrije Universiteit Brussel, Brussels, Belgium; 5 Spenshult Research and Development Center, Halmstad, Sweden

**Keywords:** arthritis, precision, ACR-1987 classification criteria, rheumatoid factor, ACPA

## Abstract

**Objective:**

Correct diagnosis of early rheumatoid arthritis (RA) is essential for optimal treatment choices. No pathognomonic test is available, and diagnosis is based on classification criteria, which can result in misdiagnosis. Here, we examined the differences between actual and misdiagnosed RA cases in a long-term cohort of patients included based on the ACR-1987 classification criteria.

**Methods:**

Patients in the BARFOT (Better Anti-Rheumatic PharmacOTherapy) cohort (n=2543) with at least four follow-up visits during the initial 5 years from enrolment were assessed, and a change in diagnosis was reported by the treating rheumatologist. The groups were analysed with respect to the individual classification criteria, antibodies to citrullinated proteins (ACPA), disease activity (DAS28) and radiographic changes from inclusion up to 2 years.

**Results:**

Forty-five patients (1.8%) were misdiagnosed (*RA-change* group). When compared to those in the *RA-change* group, the patients who kept their diagnosis (*RA-keep*) were more often RF positive (64% vs 21%, p<0.001) or ACPA positive (59% vs 8%, p<0.001). They were also more likely to fulfil more than four ACR-1987 criteria (64% vs 33%, p<0.001) and to have radiographic changes at inclusion (*RA-keep* 27% vs *RA-change* 12%, p=0.04). The groups had a similar evolution of DAS28 and its components as well as of radiological joint destruction.

**Conclusion:**

Diagnosis of RA according to the ACR-1987 criteria had a high precision in this long-term cohort. A diagnosis of RA should be re-evaluated in patients who do not fulfil more than four ACR-1987 criteria especially in patients negative for RF.

## Background

Rheumatoid arthritis (RA) is a systemic chronic inflammatory disease that can lead to lifelong disability. The diagnosis is based on typical symptoms, laboratory markers and radiographic findings. Classification criteria, but not diagnostic criteria, have been developed over the years to facilitate clinical studies, however there is no gold standard diagnostic test for RA. Using the ACR-1987 criteria,^1^ both sensitivity (the likelihood to test positive for disease if someone truly has RA) and specificity (likelihood to test negative test if someone is truly free from RA) in early RA were 77%.^2^ The corresponding percentages for the more recent 2010 EULAR/ACR classification criteria^3^ were 82% and 61%, respectively.^4^ Thus, both sets of criteria confer a risk of misclassification, where the more sensitive 2010 EULAR/ACR criteria lead to a slight decrease in specificity that increases the possibility of misclassification of non-RA patients.

The 2010 EULAR/ACR classification criteria,^3^ which focus on features at early stages of the RA disease that are associated with risk of developing persistent and/or erosive disease, have been used for a decade. These criteria put emphasis on the presence of autoantibodies, ie, anti-citrullinated protein antibodies (ACPA) and antibodies to immunoglobulins (rheumatoid factor; RF), elevated inflammatory variables and small joint involvement. A consequence is that patients with small joint arthritis and high titres of ACPA and/or rheumatoid factor (RF) are identified, whereas to fulfil the newer classification criteria, patients with seronegative RA need to present with higher degree of inflammation and more extensive joint engagement compared to the ACR-1987 criteria.^5^ The changes between different classification criteria have and will have impact on the phenotype of RA, its prognosis as well as on treatment choices.

However, there is still extensive epidemiological and clinical trials literature published using the ACR-1987 criteria. Since the diagnostic properties of the ACR-1987 criteria are better for identifying patients with established than early RA, it is of interest to know the proportion of early patients who kept their diagnosis of RA during long-term follow-up. To this end, we investigated the performance of the ACR-1987 criteria in the early RA cohort BARFOT (Better Anti-Rheumatic PharmacOTherapy) encompassing about 2800 patients followed from diagnosis up to 15 years.

## Patients and Methods

The subjects in this study were part of the Swedish BARFOT cohort^6^ that comprised 2838 patients with early RA enrolled during from 1992 to 2006, who fulfilled at least four of the seven ACR-1987 criteria and had symptom duration ≤12 months. Follow-up time was scheduled to 15 years. Over time, the diagnosis for a small proportion of the patients was changed by the treating rheumatologists to another rheumatic disease. In the current study, BARFOT patients with at least four clinical visits over the initial 5 years of follow-up were included, n=2543. The patients were divided into two groups, those who kept their diagnosis (RA-keep) throughout the follow-up and those who changed their diagnosis (RA-change). Patients in the RA-change group were excluded from the BARFOT cohort as soon as they were re-diagnosed.

### Statistical Methods

Median comparisons between RA-keep and RA-change were assessed with Mann–Whitney *U*-test for continuous clinical variables (age at first symptom, Disease Activity Score (DAS28), DAS28 components, radiographic measurements), and Chi-square (respectively Fisher’s exact for small categories) test was used to compare categorical parameters (smoking, gender, RF, fulfilment of classification criteria). For medication class at inclusion, we have additionally calculated 95% CI for the proportions to identify the exact category where the two groups differ from each other. The evolution of continuous clinical outcomes over time was assessed over 0–24 months of follow-up and we limited the observation time at 24 months to ensure good data completeness. Differences between the two groups were evaluated by means of linear repeated measure models. If a significant difference was found, a second model was fitted for the interval 3–24 months with adjustment for the baseline measurement to study whether the difference in trends could be explained by existing baseline differences. The time in the study from inclusion until date of exclusion due to relocation, refusal to continue participation, death or end of study, ie, 31 December 2019, whichever came first, was examined for the two groups with Kaplan–Meier survival analysis. In addition, for the patients within the RA-change group, the date of censoring was the date when they were re-diagnosed with another rheumatic disease. The difference in these survival curves was assessed with the Log rank test. All statistical analyses were conducted with SAS 9.4 (SAS Institute Inc., Cary, NC, USA).

### Ethics

The study complied with the Declaration of Helsinki and was approved by the Regional Ethical Review Boards at Lund University (LU 398–01, LU 368–94), Karolinska Institute (KI 02–075, T2016/297-31/1), University of Gothenburg (Gbg 88–94 and Gbg Ö 282–01) and Linköping University (LI 01–263 and LI 02–075), respectively. Informed, written consent was obtained from the participants before enrolment. All personal data was pseudonymized and handled in accordance with the Data Protection Act.

## Results

Of the 2543 patients, 45 (1.8%) belonged to the RA-change group and 2498 to the RA-keep group. The subsequently new diagnoses in the change group were psoriatic arthritis (20%), systemic lupus erythematosus (SLE) (8.9%), osteoarthritis (OA) (6.7%) as well as ankylosing spondylitis, fibromyalgia, gout, mixed connective tissue disease (MCTD), polymyalgia rheumatica, polymyositis, scleroderma, each in a proportion of 2.2%. Additionally, 49% had other unspecified diseases that simulated RA at inclusion or an inactive disease that did not require the care of a rheumatologist.

Baseline characteristics are shown in Table 1. At inclusion, the RA-change group had significantly shorter symptom duration before diagnosis, lower median DAS28, lower erythrocyte sedimentation rate (ESR) and a smaller proportion were ever-smokers compared to the RA-keep group (Table 1). Moreover, a lower proportion of patients in the RA-change group were positive for RF and ACPA and had a lower erosion score compared to the RA-keep group. A significantly lower proportion of the RA-change group received treatment with either glucocorticoids (GC), DMARDs or combination therapy both at inclusion (Table 1) and at 2 years’ follow-up (Supplementary Table 1) as indicated by non-overlapping confidence intervals.

**Table 1:** Baseline Characteristics for the RA-Change and RA-Keep Groups

	RA-Change Group	RA-Keep Group	p-value^a^
Number of participants, N (%)	45 (1.8)	2498 (98.2)	-
Female, N (%)	29 (64)	1687 (68)	0.63
Ever smoker, N (%)	18 (43)	1464 (60)	**0.03**
Age at first symptom, years	65 (46–72)	58 (47–69)	0.27
Symptom duration, months	5 (3–6)	6 (4–9)	**<0.01**
DAS28	4.8 (3.8–5.5)	5.3 (4.5–6.1)	**0.002**
Global health (VAS), mm	39 (17–60)	47 (25–65)	0.18
Swollen joint count	11 (5–16)	10 (6–14)	0.96
Tender joint count	5 (2–13)	7 (3–12)	0.17
ESR, mm	20 (9–30)	30 (17–50)	**<0.001**
RF positive^b^	9 (21%)	1582 (64%)	**<0.001**
ACPA positive^b^	2 (8%)	1100 (59%)	**<0.001**^c^
**Joint destruction**			
Total mSHS	0 (0–4)	1 (0–6)	0.11
JNS	0 (0–3)	0 (0–4)	0.23
ES	0 (0–0)	0 (0–2)	**0.03**
**Treatment**			**<0.001**^d^
No treatment (no GC, no DMARDs)	14 (31%, (16%, 47%))	279 (11%, (10%, 13%))	*S*
No DMARDs	5 (11%, (4%, 24%))	240 (10%, (9%, 11%))	*ns*
Methotrexate	14 (31%, (18–47%))	1123 (45%, (43–47%))	*ns*
Other synthetic DMARDs	12 (27%, (14–40%))	797 (32%, (30–34%))	*ns*
Combination therapy	0 (0%)	47 (1.5%, (1.4–2.4%))	*ns*
Biologic DMARDs	0 (0%)	12 (0.5%, (0.3–0.8%))	*S*

**Notes**: Statistics are presented as number (percentages with associated 95% confidence intervals) for categorical variables and median with percentile (P25–P75) for continuous variables. ^a^P-values for differences between the two groups are given according to the Mann–Whitney test for continuous variables and the Chi-square test for categorical variables. P-values that were significant at a level <0.05 are presented in bold. ^b^The number of participants with available information for RF and ACPA were 2533 and 1878, respectively. ^c^P-value given according to Fisher’s exact test. ^d^P-value given according to Chi-square test for a 2×6 comparison. Categories where the two groups differ significantly are indicated as *S*, otherwise non-significant (*ns*). Additionally, 59 patients in the RA-keep group that received biologicals or combination therapy are not included in the statistical test due to lack of a comparison category in the RA-change group.

**Abbreviations**: N, Number; DAS28, Disease Activity Score calculated on 28 joints; VAS, visual analogue scale; ESR, erythrocyte sedimentation rate; RF, rheumatoid factor; mSHS, modified Sharp van der Heijde Score; JSN, joint space narrowing; ES, erosion score; ACPA, anti-citrullinated protein antibodies; GC, glucocorticoids; DMARDs, disease-modifying antirheumatic drugs.

**Figure 1:**
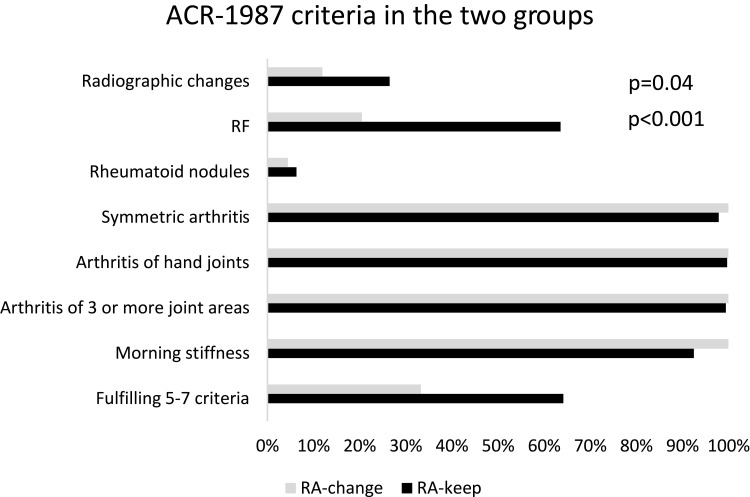
Distribution of ACR-1987 criteria, and fulfilment of 5–7 criteria, for RA-change group (grey bar) and RA-keep (black bar). P-values given according to the Chi-square test.

Of the seven stated classification criteria for ACR-1987, the two groups differed significantly at inclusion regarding RF positivity (Table 1) and radiographic changes (RA-change 12% vs RA-keep 27%, p=0.04) (Figure 1). A higher proportion of patients in the RA-change group fulfilled only 4 criteria (RA-change 67% vs RA-keep 36%, p<0.001) and a higher proportion of those in the RA-keep group filled 5–7 criteria (RA-change 33% vs RA-keep 64%). ACPA was only analysed in approx. 70% of the patients, and data were not missing at random: in the RA-change group data were missing for 47% (n=21) and for the RA-keep group 26% (n=644). It is worth noting that a significantly higher proportion of ACPA positive patients belonged to the RA-keep group (60%) than RA-change (8%) (p<0.001), which should be interpreted with caution.

**Figure 2:**
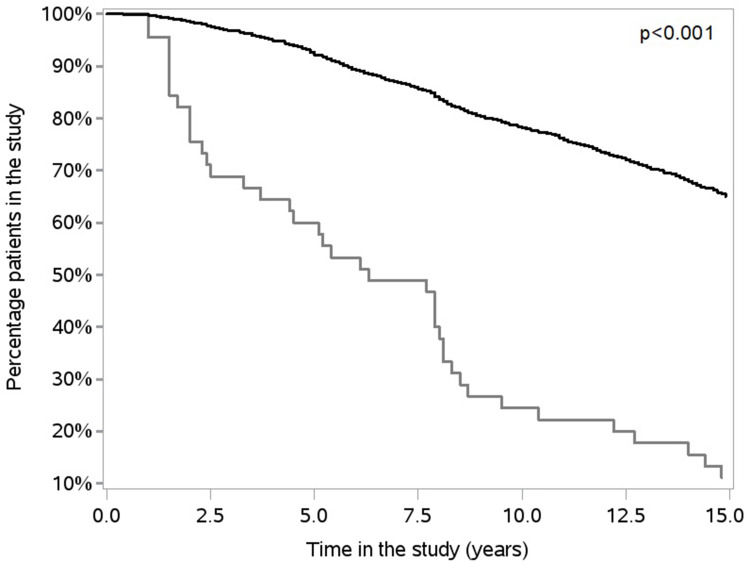
Percentage of patients in the study over time for the RA-change (bottom line) and RA-keep (top line) groups, assessed by Kaplan–Meier curves and p-value given according to the Log rank test.

Time in the study for the RA-change was significantly shorter compared to the RA-keep group (p<0.001) (Figure 2). As also shown in Figure 2, 30% of the RA-change group was re-diagnosed within 2.5 years from the initial diagnosis, and consequently 70% of these patients were still considered to have RA after 2.5 years. Thus, more than 50% of the RA-change group was still in the study 5 years after inclusion.

**Figure 3:**
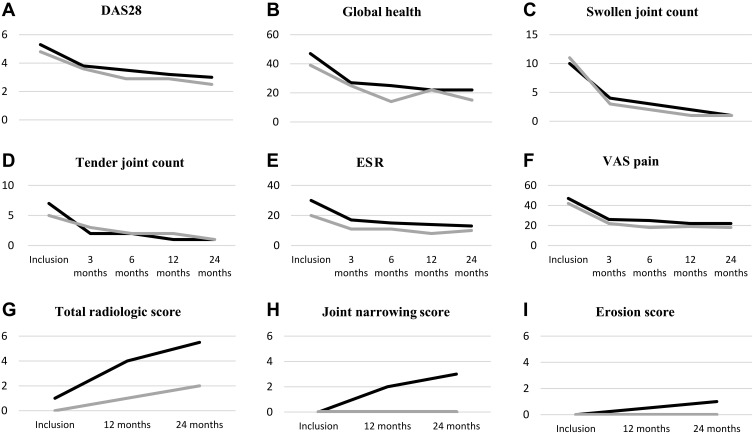
Median value for DAS28 together with its components (**A**–**E**), and VAS pain (**F**) over 2 years of follow-up. Median value for total radiological score (mSHS) (**G**) and its components joint space narrowing (JSN) (**H**) and erosion score (ES) (**I**) over 2 years of follow-up. RA-change group: grey line, RA-keep group: black line.

**Abbreviations**: DAS 28, Disease Activity Score; ESR, erythrocyte sedimentation rate, VAS, visual analogue scale; mSHS, modified Sharp van der Heijde score.

Due to a low number of remaining patients in the RA-change group, disease activity and radiographic progression were analyzed only during the first 2 years. The two groups had a similar evolution of the DAS28 and its components and VAS pain (Figure 3A–F), with a substantial decrease from inclusion to 3 months. Only ESR differed significantly between the groups, with RA-change being 5.7 units lower than RA-keep group over time (p=0.02). Analyzing the evolution of DAS28 from 3 to 24 months, with adjustment for inclusion ESR, the group difference was no longer significant suggesting that the difference over time could be explained by the baseline group difference.

Total radiological joint destruction, assessed by modified Sharp van der Heijde score, increased over time in both groups, with RA-change group having 1.8 units lower score compared to RA-keep, however this difference was not significant (Figure 3G). Joint space narrowing and erosion score (Figure 3H–I) did not differ significantly over time between groups.

## Discussion

In this study, we found that 1.8% of a large RA cohort with cases included based on ACR-1987 criteria changed their diagnosis during 15 years of follow-up. A lower proportion of the patients in the RA-change group fulfilled >4 criteria, had lower ESR, were less often RF- and/or ACPA-positive and less likely to have developed joint erosion at diagnosis.

Both early and correct RA diagnosis is important for the patient, as a mistake may result in incorrect treatment that has an impact on the disease itself, associated comorbidities, quality of life and socioeconomic factors. Differential diagnoses include rheumatological conditions such as psoriatic arthritis, OA and SLE. The diagnosis of rheumatic diseases is most often based on classification criteria and pathognomic markers are scarce. Systemic inflammatory diseases such as SLE and overlap syndromes such as MCTD pose diagnostic challenges where the first diagnosis could be revised as more symptoms or signs appear. Patients with both these diseases often present with positive RF and arthritis. Not surprisingly, we find both these diseases among those misdiagnosed. Musculoskeletal symptoms resembling RA can also appear in other diseases such as cancer,^7^ where in particular RF positivity was related to increased mortality.^8^ Other diseases that can mimic RA include storage diseases, eg, hemochromatosis^9^ and endocrine diseases eg, diabetic osteoarthropathy.^10^ Also, chronic infections, eg, in Whipple’s disease or viral infections such as Chikungunya or Parvovirus B19,^11^,^12^ are important differential diagnoses that can present with symptoms and clinical findings very similar to those of RA.

The most common differential diagnosis to RA is OA^13^ that share a number of risk factors, eg, female gender and age, as well as a range of early clinical symptoms.^14^ We expected therefore that OA would be the most common reason for re-diagnosis but found that it was psoriatic arthritis. As only 1.8% of the patients were misdiagnosed, it is likely that the experienced rheumatologists who included the patients in the study identified the differences in symptoms as well as the lack of systemic inflammatory markers in OA but present in psoriatic arthritis. It is, of course, of importance to distinguish between RA and OA as early treatment with immunosuppression has been shown to be beneficial in RA, reviewed in reference,^15^ whereas in OA such treatment has no effect and might cause side effects. In the present study, a significantly lower proportion of the RA-change patients received treatment at inclusion as well as after 2 years compared to those in the RA-keep group. The reasons are unclear, but the lower levels of ESR in the RA-change group might contribute, albeit the disease activity measured as DAS28 was similar in the two groups.

Although the ACR-1987 criteria have rather low sensitivity and specificity in early RA, the low proportion of patients, who received another diagnosis in the BARFOT cohort, indicates that these criteria, applied by experienced rheumatologists in the setting of a structured protocol, are valid from a diagnostic point of view. This assumption is further strengthened by the extensive literature derived from studies in which these criteria were used. Addition of ACPA increases the diagnostic potential and could facilitate an early diagnosis.^4^ However, also in the recent NORD-STAR cohort,^16^ based on the 2010 EULAR/ACR criteria, symptom duration was approx. 6 months the same as in BARFOT.^6^ It would have been of interest to validate the ACR-1987 criteria versus the 2010 EULAR/ACR criteria in BARFOT. Unfortunately, this was not possible since the information about ACPA and RF titres as well as the distribution of swollen and tender joints were lacking in BARFOT. Analysing the presence of ACPA, we found a significantly higher proportion of positive patients in the RA-keep group and as expected, those positive showed a worse radiological progression. However, due to the low number of participants in the RA-change group, together with ACPA data not missing at random this finding should be interpreted with caution. The likelihood of misdiagnosis seems to have increased with the 2010 EULAR/ACR criteria as the patients are diagnosed very early on: in one study, as much as 10% of the patients initially diagnosed with RA were given a definite alternative diagnosis.^17^ However, the 2010 criteria perform much better in the early identification of RA patients. A study including over 500 patients from two established cohorts who fulfilled the 1987 criteria at 1 year but not at inclusion found that when applying the 2010 criteria 57% and 75% of these patients, respectively, would have been identified as RA already at inclusion.^18^ Among the ACPA-positive patients, the early detection based on 2010 criteria was approx. 92%. Among the ACPA-negative patients, the detection varied between 25% and 51%, demonstrating that the 2010 EULAR/ACR criteria are better than the ACR-1987 at an early identification of RA, especially in autoantibody-positive patients. In line with this, a much higher proportion of patients in NORD-STAR^16^ compared to BARFOT was positive for both RF and ACPA and presented with a worse global health and more pronounced radiological destruction^19^ (Supplementary Table 2). However, the 2010 EULAR/ACR criteria do not perform equally well in autoantibody negative patients.^5^

Study limitations include a small number of participants in the RA-change group from a statistical point of view and lack of a structured protocol for identification of other inflammatory diagnosis. The strength of the study is structured protocols at inclusion and throughout the long-term follow-up.

## Conclusions

The diagnosis of RA can be challenging, especially in early disease. However, fulfilment of more than four ACR-1987 criteria of which one is RF-positivity increases the likelihood of a correct diagnosis. Thus, if these conditions are not met, the diagnosis should be continuously re-evaluated. A diagnosis of RA according to the ACR-1987 criteria has a high diagnostic precision in patients with symptom duration <1 year, which was confirmed in this long-term follow-up study.
